# Atraumatic Back Pain Due to Quadratus Lumborum Spasm Treated by Physical Therapy with Manual Trigger Point Therapy in the Emergency Department

**DOI:** 10.5811/cpcem.2019.4.42788

**Published:** 2019-05-29

**Authors:** Casey Grover, Kory Christoffersen, Lindsay Clark, Reb Close, Stephanie Layhe

**Affiliations:** Community Hospital of Monterey, Department of Emergency Medicine, Monterey, California

## Abstract

Manual trigger point therapy is effective for treating myofascial pain, yet it is not frequently used in emergency department (ED) settings. A 42-year-old female presented to the ED with atraumatic back pain. Her pain was thought to be myofascial, and we obtained a physical therapy consultation. Diagnosing the patient with quadratus lumborum spasm, the physical therapist treated her in the ED using manual trigger point therapy, and completely relieved her pain without requiring any medications. Manual trigger point therapy can provide non-opioid pain relief in ED patients, and physical therapists can apply this technique effectively in the ED.

## INTRODUCTION

Given the current epidemic of opiate use, addiction, and death from overdose in the United States,^1^–^3^ non-opioid therapies to treat pain are needed to avoid exposing patients to the risk of opioid dependence. We discuss the use of manual trigger point therapy by emergency providers and physical therapists in the emergency department (ED), as an underused non-opioid treatment for pain management in the ED.

## CASE REPORT

A 42-year-old female presented to the ED with left-sided back pain upon waking up in the morning. She reported that the pain was located in the left posterior lower ribs, about the inferior portion of the left scapula. The patient denied associated fevers, trauma, or rashes. She stated that her pain was worse with movement and taking a deep breath, and when reaching her left arm across the right side of the body. She denied any associated numbness or weakness. Her past medical history was notable for hypertension, but she could not recall the name of her anti-hypertensive medication. She had no allergies and had no other significant past medical or social history, although she did smoke e-cigarettes.

On examination, her vital signs were within normal limits. There was no hypoxia, tachypnea, or tachycardia. Pertinent physical exam findings revealed that the patient was experiencing moderate distress secondary to pain. She had a normal cardiac and pulmonary auscultation, and her skin was normal. Neurologically, her strength and sensation were intact in the upper and lower extremities. On musculoskeletal examination, the provider noted pain with forced adduction of the left arm across the body, and back exam was notable for tenderness in the left paravertebral muscles of the thoracic spine. The provider initially reported concern for pulmonary embolism, which was excluded with the Pulmonary Embolism Rule-out Criteria, as well as concern for pneumothorax and occult rib injury. A chest radiograph with dedicated left-sided rib views revealed no acute abnormality.

Given that the patient’s pain appeared to be myofascial in origin, a physical therapy consultation was obtained. The physical therapist was specifically trained in myofascial manipulation and trigger point release. The patient was diagnosed by the physical therapist with muscular spasm of the left quadratus lumborum muscle, and was treated with manual trigger point therapy, which completely released the spasm in the muscle. Upon re-assessment by the emergency physician (EP), the patient was pain free and had not required any medication while in the ED. She was discharged with topical diclofenac to be used in case the spasm re-occurred.

## DISCUSSION

Manual trigger point therapy is a technique that can be used by healthcare providers from multiple training backgrounds. It involves assessment of a patient in pain for myofascial trigger points, followed by the use of manual techniques to de-activate the trigger point, which results in a decrease in, or resolution of the patient’s pain. A myofascial trigger point is a hard, palpable nodule in a tight band of muscle that is hyperirritable and painful. Such trigger points often have multiple contraction knots within the muscle, and are tender on examination.^4^ While several therapies are available for management of myofascial trigger points, manual therapy may be used; it involves the use of a provider’s hands to provide treatment.

Manual therapy can be defined as application of an accurately determined and specifically directed force to the body to address dysfunction in joints, connective tissue, or muscle. Techniques may include trigger point pressure release, or trigger point compression.^5^–^6^ At our institution, physical therapists have been trained in manual trigger point therapy (course: Myopain Seminars, Bethesda, Maryland), and are available to evaluate and treat ED patients with myofascial pain. An important detail is that manual trigger point therapy involves providing treatment with the provider’s hands alone, as opposed to a technique such as trigger point injection or trigger point needling, both of which involve the use of a needle.

While trigger point injections may be used by EPs,^7^ they may not be aware of the technique or know how to perform it. Additionally, many patients express a phobia of needles and are not willing to receive an injection; however, they are open to other treatments, which, in the case of severe muscle spasm, may lead to the use of oral opioids or benzodiazepines. Manual trigger point therapy is an effective means of treating muscle spasm without using needles or potentially addictive medications, and therefore represents a novel option for non-opioid ED pain management.

Manual trigger point therapy may be performed by healthcare providers from a variety of training backgrounds, including physical or occupational therapists as well as physicians. As EPs are often busy with multiple critical patients, physical therapists are an excellent option to provide treatment with this technique in the ED. At our institution, manual trigger point therapy has been performed by physical therapy in the ED since March 2018. Physical therapists are available to provide this treatment between 8 am and 4 pm, seven days per week. When this service is needed outside of these hours, the EP can provide a trigger point injection (if the patient is willing), use medication, or use a transcutaneous electrical nerve stimulation unit to provide relief. Two osteopathic physicians in our department provide treatment similar to manual trigger point therapy with osteopathic manipulation. In one case, a patient who presented to the ED in the late evening had severe muscle spasm and pain that could not be controlled in the ED with medication. The patient was admitted to the observation unit for physical therapy consultation and treatment with manual trigger point release the next morning, which provided good relief of her pain and spasm.

**CPC-EM Capsule**

What do we already know about this clinical entity?

Pain is a common presentation to the emergency department (ED), and non-opioid treatments are desirable. Trigger point therapy can provide non-opioid relief.

What makes this presentation of disease reportable?

This is the first report of a physical therapist successfully providing manual trigger point therapy in the ED to relieve pain.

What is the major learning point?

Emergency providers should consider trigger point therapy to provide non-opioid pain relief.

How might this improve emergency medicine practice?

Using trigger point therapy in the ED, including when provided by physical therapists, can improve pain relief without opioids.

An informal survey of our EPs (n = 12) revealed that 100% of them agreed the treatment was useful for treating pain, and 100% felt that the technique was an effective intervention to reduce the use of opioids in the ED. A similar informal survey of the physical therapists trained in the technique at our hospital (n = 9) revealed that 100% of them also felt that the treatment was useful for treating pain.

To the best of our knowledge, this is the first report of using manual trigger point therapy to treat muscle spasm by physical therapists in the ED. While this intervention may have been performed in this way at other institutions, we chose to report our case as it was so successful at relieving pain without the use of medication, and may encourage further use of this technique and future research on the topic.

## CONCLUSION

Manual trigger point therapy is an inexpensive and effective way to treat myofascial pain and can be used in the ED, particularly when it is advisable to avoid opioids or other sedating medications. If EPs are unable to perform the treatment, physical therapists can be easily trained in the technique and are effective in using it to treat pain in ED patients.
